# Meeting the Health Needs of Migrant Workers Affected by the Tsunami

**DOI:** 10.1371/journal.pmed.0020176

**Published:** 2005-06-28

**Authors:** David Wilson

## Abstract

David Wilson of Médecins Sans Frontières, Thailand, discusses how the tsunami excacerbated the health status of Burmese migrants.

## Teaser

Burmese migrant workers in Thailand are a vulnerable group

**Figure:**
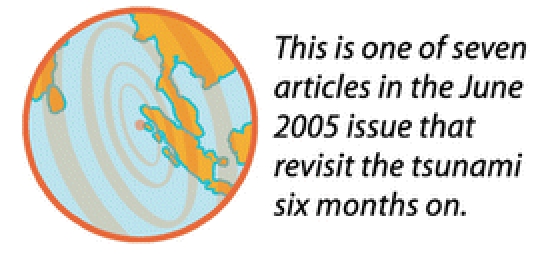


**Figure 1:**
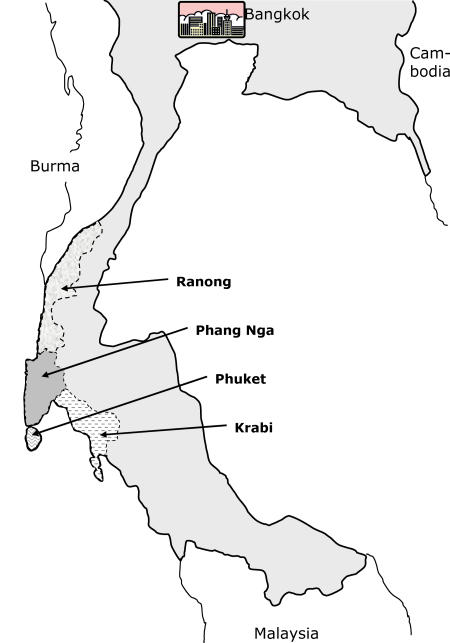
Four Provinces of Southern Thailand Most Severely Affected by the Tsunami (Source: Thai Ministry of Interior Department of Disaster Prevention and Mitigation)

Following last December's tsunami, Thailand reported 10,385 deaths and missing persons among Thais and foreign tourists, 80% of them in Phang Nga province (Figure 1). These reported tsunami deaths do not include Burmese migrant workers, which one local organisation estimates as being as many as 1,000 individuals [1].

Medical relief to Thai nationals and Western victims was well organised [2], but Burmese migrants could not always benefit. This article briefly highlights how the tsunami affected this vulnerable group, exacerbating pre-existing difficulties in access to health care, and how Médecins Sans Frontières (MSF) is responding.

## Migrant Workers in Thailand

Prior to the tsunami, 30,572 registered migrants, 98.6% of them Burmese, worked in fisheries, coastline construction, agriculture, and tourism in Phang Nga province [3].

Nongovernmental organisations estimate there are, in addition, an equal number of illegal migrants, who are in effect undocumented refugees. Migrant workers are vulnerable to exploitation, migration being largely unregulated and partially criminalized [4].

## Access to Health Care

Registration requires participation in a health insurance scheme, giving registered migrants rights of access comparable to those of Thai citizens to Thailand's low-cost health-care system. Undocumented migrants cannot legally access health care. However MSF's observation is that sick Burmese, if they can speak Thai or are accompanied by a translator, do receive appropriate health care, whether registered or unregistered. Unregistered patients without money may be treated free for common complaints. Hospitals also subsidise more specialised treatment but approach the employer for a further contribution.

In practice, Burmese migrants rarely access even basic preventative health services such as vaccination. Very few married women we have met use any form of contraception, even if they do not want to become pregnant. Deliveries are at home, in poor conditions, often under the supervision of a traditional birth attendant.

Burmese to whom we spoke listed the following constraints to accessing adequate health care: (1) being unable to speak and read Thai, (2) not knowing what health services are available, (3) having fears about security, and (4) having no money for transport costs.

## The Impact of the Tsunami

The tsunami killed employers and destroyed construction sites, leaving many migrants without work, and many returned to Burma or relocated to other areas of southern Thailand, either for fear of another tsunami or to find work. Police began arresting and deporting illegal migrants, but also registered migrants who may have lost their documentation [5]. MSF has met members of families who were split up in the deportation process.

Following a request to the Ministry of the Interior by international governmental and nongovernmental organisations on January 26th, these arrests and deportations have stopped, but a climate of fear remains—one month after the tsunami we met two Burmese with tuberculosis who had stopped treatment for fear of arrest whilst travelling to hospital.

Reconstruction in Phang Nga is proceeding slowly compared with other provinces [6], because of delay in distribution of government assistance [7], but as the reconstruction speeds up, further migrant workers are likely to arrive and problems to exacerbate. Community organisations already tell us of increasing numbers of migrant workers in Phang Nga, of whom more than half are unregistered.

## Health-Care Priorities for Migrant Workers: High Levels of STIs

The need for post-tsunami assistance will be long term [8]. After supporting the Thai government's initial relief effort, MSF began to focus on strengthening the health-care system for migrant workers in Phang Nga. We had early contact with a community of 80 Burmese and Thai construction workers, at which point we referred two Burmese women with suspected AIDS (later confirmed) and one with gonorrhoea and pelvic inflammatory disease to hospital.

High levels of sexually transmitted infection (STIs) and HIV amongst Burmese migrants are unlikely to be confined to this site. Syphilis serology was positive (RPR+) in a high 6% of tested antenatal patients in MSF clinics in Yangon, Burma's capital, in 2004, having fallen from still higher rates in previous years following intensive STI prevention and treatment efforts.

STI rates are low in Thailand, with syphilis serology being positive (VDRL+) in only 0.18% of tested antenatal patients nationwide [9].

The low rate in Thailand is not a cause for complacency. Local nongovernmental organisations and migrant workers tell us that before the tsunami there were many sex workers along the coastal areas serving both migrant and host communities. Where destruction from the tsunami was less, we have seen that this behaviour continues. Lack of knowledge about STIs is a problem. Our first health education session for Burmese migrants revealed that none had ever used a condom; only four men knew anything about STIs: two of these had had an STI previously.

## Conclusion

To improve knowledge and understanding of health by migrant workers, and their access to health care, we must first learn more about both the migrant and host communities and how they interact. Many migrants are undocumented, and some remain in hiding. We do not have a full appreciation of communities' own perspectives on health priorities. Working in this environment will require being sensitive to the concerns of other stakeholders, including employers who will want to know what a nongovernmental organisation is doing with their workforce.

## Abbreviations

MSF: Médecins Sans Frontières
STI: sexually transmitted infection

## References

[pmed-0020176-b1] Saydana Tsunami (2005). Situation report No. 1: Burmese migrant workers in Thailand. http://www.saydanatsunami.org/report.php?#report1.

[pmed-0020176-b2] Wacharong C, Chukpaiwong B, Mahaisavariya B (2005). After the tsunamis: Phang Nga, Thailand. The Lancet.

[pmed-0020176-b3] International Organization for Migration, United Nations High Commission for Refugees, UNIFEM (United Nations Development Fund for Women), United Nations Office of the High Commission for Human Rights, World Bank Technical Assistance Mission Report: Joint Tsunami Migrant Assistance Mission to the Provinces of Krabi, Phang Nga, Phuket and Ranong.

[pmed-0020176-b4] International Organization for Migration (2003). Migration in South East Asia. http://www.iom-seasia.org/index.php.

[pmed-0020176-b5] Saydana Tsunami (2005). Situation report No. 2. http://www.saydanatsunami.org/report.php?#report2.

[pmed-0020176-b6] McGeown K (2005). Thailand's tsunami-hit tourism.

[pmed-0020176-b7] Srimalee S (2005 April 4). Tsunami: 100 days on, firms still await financial aid. The Nation, Business p.

[pmed-0020176-b8] Walker P, Wisner B, Leaning J, Minear L (2005). Smoke and mirrors: Deficiencies in disaster funding. BMJ.

[pmed-0020176-b9] Thai Ministry of Public Health.

